# Supplementary material for: Complexome profiling of the Chlamydomonas psb28 mutant reveals TEF5 as an early PSII assembly factor
Source: Plant Cell. 2025 Mar 17;37(6):koaf055. doi: 10.1093/plcell/koaf055 (PMC12164590; doi:10.1093/plcell/koaf055)
Supplement: koaf055_Supplementary_Data [file koaf055_supplementary_data.zip › psb28_interactive.zip!/plots/intensity/qualityPlots/peptides/FMG1_Cre18.g750047.t1.1.html]

### Cre18.g750047.t1.1

Trivial name: FMG1  
SeqCoverage [median]: 12.20  
Mass: 447.50 kDa  
  
 

| Sequence | MissedCleavage | Mass [Da] | wtIntensity | muIntensity | Score |
| --- | --- | --- | --- | --- | --- |
| AAGSDAASSPAFAR | 0 | 1277.60 | 17781.78 | 48270.80 | 310.53 |
| AFDVASATPQSIVSATAVGPTR | 0 | 2145.11 | 6112.39 | 18390.16 | 119.01 |
| AGAAVTQLGK | 0 | 914.52 | 4350.05 | 11719.55 | 166.61 |
| AGSSSGPLYGPGAAVTIVPK | 0 | 1827.97 | 18221.17 | 58586.58 | 166.09 |
| AGTATGLVYTSK | 0 | 1167.61 | 763.18 | 5951.99 | 155.11 |
| ASQTMLK | 0 | 777.41 | 6913.09 | 22876.17 | 166.05 |
| ASQTTLK | 0 | 747.41 | 8506.47 | 12314.70 | 174.69 |
| GTVLTVK | 0 | 716.44 | 7158.93 | 30895.56 | 139.28 |
| GVALNTASPIYK | 0 | 1232.68 | 19012.16 | 59638.35 | 261.03 |
| IATPATAR | 0 | 799.46 | 14854.53 | 24569.89 | 110.81 |
| ILSQATLGLK | 0 | 1042.64 | 11231.85 | 51048.01 | 148.52 |
| LASGSEFANGDQVNIHATTNTGTK | 0 | 2432.16 | 0.00 | 6568.06 | 119.34 |
| LGSPIAVADAR | 0 | 1068.59 | 12082.58 | 28710.20 | 162.80 |
| LGTGASAAYYVPLPSSAAVIPTIASAK | 0 | 2575.39 | 4983.85 | 0.00 | 84.68 |
| LNAAFLTSASTIVAK | 0 | 1505.85 | 11711.44 | 9540.91 | 207.47 |
| LTVNAAQATTTATSYLK | 0 | 1752.93 | 664.96 | 12972.10 | 143.94 |
| LYDYAPATGASTAEITR | 0 | 1798.87 | 9504.53 | 47074.07 | 165.51 |
| RSPVVIEPR | 1 | 1051.61 | 3244.34 | 15932.68 | 90.83 |
| SAGTYAPGDTVNVVNGQSTLR | 0 | 2106.03 | 742.54 | 2290.70 | 121.44 |
| SAQTALTVGTTK | 0 | 1176.63 | 7255.80 | 17901.06 | 194.17 |
| SGQSLLK | 0 | 731.42 | 12276.35 | 33617.54 | 204.65 |
| SGQSLLKADSTTAANR | 1 | 1618.83 | 1243.08 | 7204.07 | 169.92 |
| SIVVAAASSTAAK | 0 | 1174.66 | 24448.86 | 78150.55 | 176.95 |
| SPVVIEPR | 0 | 895.51 | 6346.96 | 25535.82 | 123.98 |
| TGNTALVGGSDPATGAPYVATTALPIR | 0 | 2570.33 | 7058.82 | 39578.13 | 135.84 |
| TTSGGVSTITVPLTTNIAIGDTVR | 0 | 2373.28 | 3653.99 | 0.00 | 63.57 |
| TVVVTLPATSTVYK | 0 | 1477.84 | 3925.12 | 8648.00 | 124.10 |
| VQLPIASDLAK | 0 | 1153.67 | 17387.47 | 80948.16 | 110.38 |
| VRILSQATLGLK | 1 | 1297.81 | 453.75 | 0.00 | 49.45 |
| VSALATGPSYVPK | 0 | 1288.70 | 23479.98 | 57281.37 | 245.76 |
| WTNTVLR | 0 | 888.48 | 0.00 | 2148.51 | 77.74 |
|  |  |  | 265370.0 | 818363.7 |  |
